# The VML method intervention effectiveness for childhood Apraxia of speech – professional and non-professional treatment

**DOI:** 10.1192/j.eurpsy.2023.693

**Published:** 2023-07-19

**Authors:** E. Vashdi

**Affiliations:** Research, Yaelcenter, Aloney Aba, Israel

## Abstract

**Introduction:**

The VML (Verbal Motor Learning) method (Vashdi, 2013, Vashdi, 2014, 2017) is an organized, structured method which includes unique evaluation, algorithmic analysis, manual techniques, motor learning principles and unique treatment principles. The VML method targets the Apraxia of speech syndrome deficits, while teaching the learner how the plan the speech movements and time the speech systems for an accurate pronunciation.

Being a multidimensional entity in nature, as a speech therapy tool developed by a physical therapist, the VML method crosses disciplinary boundaries of agreement and proposing new policies for treatment. The speech tool serves other professions then speech therapists and is also given to parents for practice and learning. The parent’s role in the therapeutic team is controversial, however, we have found that parent’s intervention in some cases can be very effective, contributing to treatment process.

**Objectives:**

The purpose of this retrospective study is to answer two major questions; What is the efficacy of the professional and non-professional VML treatment.

**Methods:**

A retrospective study was conducted analyzing 900 evaluations of which 136 longitudinal treatment processes of children diagnosed with CAS or suspected CAS, were extracted. The participants contacted the early age intervention clinic for VML speech evaluation on their own will, and were examined by a VML expert. The data was collected over the years 2006-2018 based on children evaluated at the clinic in Israel. A set of variables based on the VML method assessment (Vashdi, 2013; Vashdi, 2014) was established for the retrospective data collection

136 speech treatment processes passed the inclusion criteria and were examined (59 professional treatment and 77 non-professional treatment

**Results:**

There were no differences between the professional and non-professional group’s baseline data. The entry syllables score for all participants was 16.32%. The average improvement in syllables for all participants was significant (33.18% , p<0.05, paired t-test). It was also found significant for the professional and non-professional treatment groups ( 45.49% and 23.75% respectively, p<0.001, t-test). Single syllables improvement was found higher for professional group (P=0.02).

**Conclusions:**

Both groups demonstrated significant improvement in the single syllables production skill, while the professional group was found significantly more effective than the home-based treatment. The findings regarding the professional group are not surprising, however support the efficacy of the VML intervention for children with severe CAS, even with one session per week of intervention. The non-professional home based treatment results were surprising and promosing for future practice.

**Disclosure of Interest:**

None Declared

